# Annotations

**Published:** 1905-12-16

**Authors:** 


### ANNOTATIONS.

### The New President of the Local Government Board.

The late Prime Minister, having just before his retirement from office nominated a Royal Commission to inquire into the working of the Poor Law, from which persons of practical experience in the treatment of the sick with the exception of a single able official are excluded, his successor has appointed as President of the Local Government Board a man who, from the expert point of view and whatever his general gifts, has no special qualifications for the post beyond his intimate acquaintance with the wants of the poor. We do not in the least complain of the inclusion of Mr. John Burns in the Cabinet. Quite the contrary. More than some of his colleagues, he has, politically speaking, earned a place, a substantial salary, and the title of Right Honourable. But since it was necessary to give him a share in the loaves and fishes, why make him chief of a department whose head should possess above all things a scientific training and large expert knowledge? Mr. Burns, who is not, of course, in any way to blame for accepting the post, would be the first to admit that these are not his strongest recommendations for the office. In the discharge of his duties he will, we fear, either have to depend largely upon the advice of the permanent officials, or to decide on his own account questions of which he has comparatively little knowledge. As there never was a time when the Local Government Board more greatly needed a head in no sense at the mercy of his subordinates, nor exposed to the risk, through possible misunderstanding of the requirements of the situation, of committing some disastrous blunder, Sir Henry Campbell-Bannerman cannot be altogether congratulated upon the precise manner in which he has recognised the services of Mr. Burns. The new President, we gladly acknowledge, has a fund of common sense, which may help him to tide over times of particular difficulty, but it seems a pity that this office was not assigned to someone more directly suited for it. We can only hope that Mr. Burns will fulfil the confident anticipations of his friends, who are of opinion that he is the right man in the right place.

### Women's Clubs and Race Suicide.

An indictment against women's clubs has been drawn up by an American lady. She cites figures in support of a contention that only one married woman in ten becomes a mother after she has identified herself with clubs for women; and she avers that only one in forty-three, during a period of eight years, has become the mother of two children under the same conditions. The General Federation of Women's Clubs affirm that there is "a complete answer" to these allegations. Without accepting the allegations as accurate we are afraid that the formation of women's clubs, even in this country, has not been altogether beneficial to the race. To some extent, no doubt, they are to be welcomed, at any rate in the interests of unmarried women who have no home ties and no one dependent upon them, either for care or for assistance. In fact, a club is often in their case a necessary convenience, which facilitates work and has other practical advantages which they rightly value. Even these advantages are not unmixed, and membership may easily mean the acquisition of habits which do not add to the charms or the physical benefit of unmarried women. But it cannot be otherwise than detrimental to family life if a married woman spends time at a club which ought to be occupied at home, whether in domestic pursuits or in the society of her children. There is an element of excitement or unrest in club life which can scarcely fail to interfere with the performance of the duties of motherhood, even if it does not have more serious consequences upon the mother. On grounds of health, and for the sake of the unborn as well as of children who require the constant attention of a mother, we certainly deprecate the development of club life for the mothers of the future.

### The Practitioner and his Price.

The position of medicine among the professions is in many respects a most anomalous one, principally because medicine and charity seem to be, and indeed are in practice, almost indissolubly linked. There is probably no other professional life the apprenticeship to which involves so much free giving of skilled opinion and attention as does that of the young resident at a large general hospital; and this is no matter for complaint on his part, since he is acquiring knowledge which may be expected to bring in ultimately a proportionate return. But the medical man may fairly claim that, when he announces the termination of his apprenticeship by embarking upon a practice, he should be regarded as a business man, following an honourable profession for a livelihood and not as a philanthropist. For, after all, most men who devote themselves to medicine are not actuated by philanthropic motives in so doing, though, to their credit, they are never slow in rendering gratuitous service. This comment is prompted by an incident lately dealt with by the *British Medical Journal*, the journal of the British Medical Association, the journal of the rights of the faculty. A medical man was summoned by the police to attend a patient suffering from the effects of an overdose of opium. The call came soon after 12 midnight, and the practitioner was detained until 8.30 A.M. He wrote to the *British Medical Journal* to ask advice as to the proper fee to be demanded of the police. The answer was—two guineas! Here, for once, the question of charity did not vitiate the argument. We are face to face with an authoritative statement from the familiar councillor of the profession that skilled medical attention (altogether apart from considerations of charity) is worth five shillings an hour, and that for an eight-hour spell commencing at midnight! Save us from our friends!

